# A novel micro-CT analysis for evaluating the regenerative potential of bone augmentation xenografts in rabbit calvarias

**DOI:** 10.1038/s41598-024-54313-4

**Published:** 2024-02-21

**Authors:** Ilan Beitlitum, Fatma Rayyan, Ariel Pokhojaev, Haim Tal, Rachel Sarig

**Affiliations:** 1 Department of Periodontology and Dental Implantology, The Maurice and Gabriela Goldschleger School of Dental Medicine, Faculty of Medicine, Tel Aviv University, Tel Aviv, 6997801 Israel; 2 Department of Oral Biology, The Maurice and Gabriela Goldschleger School of Dental Medicine, Faculty of Medicine, Tel Aviv University, Tel Aviv, 6997801 Israel; 3 Shmunis Family Anthropology Institute, the Dan David Center for Human Evolution and Biohistory Research, Faculty of Medicine, Tel-Aviv University, Tel Aviv, 6997801 Israel

**Keywords:** Guided bone regeneration (GBR), Grafting materials, Biomaterials, Bone regeneration, Critical size defects, Micro-CT, Dental biomaterials, Resorption, Dentistry, Resorption

## Abstract

Guided Bone Regeneration is a common procedure, yet, as new grafting materials are being introduced into the market, a reliable evaluation method is required. Critical size defect in animal models provides an accurate simulation, followed by histological sections to evaluate the new bone formation. However, histology is destructive, two-dimensional and technique-sensitive. In this study we developed a novel volumetric Micro-CT analysis to quantify new bone formation characteristics. Eight adult female New Zealand white rabbits were subjected to calvarial critical-size defects. Four 8 mm in diameter circular defects were preformed in each animal, to allow random allocation of four treatment modalities. All calvarias were scanned using Micro-CT. Each defect was segmented into four equal parts: pristine bone, outer, middle, and inner. Amira software (v. 6.3, www.fei.com) was used to calculate the new bone volume in each region and compare it to that of the pristine bone. All grafting materials demonstrated that new bone formation decreased as it moved inward. Only the inner region differed across grafting materials (p = 0.001). The new Micro-CT analysis allowed us to divide each defect into 3D regions providing better understanding of the bone formation process. Amongst the various advantages of the Micro-CT, it enables us to quantify the graft materials and the newly formed bone independently, and to describe the defect morphology in 3D (bi- vs. uni-cortical defects). Providing an insight into the inner region of the defect can better predict the regenerative potential of the bone augmentation graft material. Therefore, the suggested Micro-CT analysis is beneficial for further developing of clinical approaches.

## Introduction

Guided Bone Regeneration (GBR) is a well-established and effective procedure^1–5^, in which a barrier membrane is placed over a biocompatible bone graft material^6^. While autogenous bone remains the gold standard for bone grafts^7^, researchers have been exploring alternative bone graft materials that can serve as scaffolds for bone growth and membrane supports^8–10^. The most commonly used materials are human-derived allografts and bovine or porcine xenografts; however, disease transmission potential risks^11,12^ and resorption characteristics limit their use. In certain regions, the usage of allografts is limited due to legal and health regulations^8,9^. To address these limitations, researchers have investigated alternative materials. One promising option is biomaterials derived from mineralizing marine organisms, particularly sea coral xenografts utilized for bone grafting purposes^13–15^.

Animal models are often utilized to explore and confirm the efficacy of new materials, with the calvaria bone defect procedure commonly performed in rodent species^5^ . The preferred species for this model are rats and rabbits and it has undergone extensive studies and is highly reproducible^4^. Moreover, the calvaria’s flat shape enables the creation of consistent defects, making it easier to compare different graft materials^4^. Critical size defect (CSD) is one of the common animal models, which was defined by Schmitz et al.^16^ as “the smallest size intraosseous wound in a particular bone and species of animal that will not heal spontaneously during the lifetime of the animal”^16^. Often, the CSD size used for animal model was 15 mm^7^. Cooper et al.^8^ suggested a different definition of CSD: ”any defect that does not heal over the duration of the study”^8^. This suggests that the classification of a defect as CSD can also depend on the research duration; for instance, an 8 mm defect may be classified as CSD in studies extending up to 12 weeks^7^.

Another consideration should be the defect morphology, In a study by Borie et al.^9^, 8 mm bi-cortical defects in rabbit calvaria failed to fully heal after 12 weeks without a bone graft^9^. It’s uncertain whether uni-cortical defects would yield different outcomes.

Histological sections are the common method for evaluating bone formation in animal models, but they have certain limitations. Variations in sampling and methodology can affect the results^12^. In regards to bone biopsy, it is crucial to note that the number of sections that can be taken is restricted and is impacted by both the area and orientation of each section^11^, For instance, it is unable to present a complete assessment of the lesion’s morphology, such as whether it is uni- or bi-cortical. It is evident that no single method can completely characterize the quality of the bone^10^ . Moreover, the process of preparation and sectioning may cause damage to the specimen and result in distortion. It is important to note that no single method can fully characterize bone quality. Thus, incorporating a digital method to assess bone formation can offer numerous advantages. Studies have proposed that Micro-CT can complement histological findings in bone research^17^; it is a non-invasive and conservative technique, enabling 3D reconstruction of the specimen^17^; additionally, it can be employed for quantitative morphometry of bone, such as volumetric and spatial distribution analysis, and many other benefits^17–19^.

The purpose of this study was to create a novel Micro-CT analysis that enables a precise 3D evaluation of bone volume and morphology across the different regions of the defect. For this purpose, we utilized CSD on a rabbit calvaria model to evaluate the effectiveness of a new Pure Coral Mineral (PCM) graft (CORBONE®, Misgav, Israel) and compare it with two commonly used Xenografts - DBBMb (Deproteinized Bovine Bone Mineral - Bio-Oss) (Bio-Oss, Geistlich Pharma AG) and DBBMc (Deproteinized Bovine Bone Mineral - Cerabone)(Cerabone, Botiss Biomaterials, Berlin, Germany)^20^. In our study, we conducted a comparative analysis between two commercially available xenograft materials. The first material, DBBMc, is prepared using a high-temperature processing technique applied to the bovine bone, while the second material, DBBMb, is prepared using a low-temperature processing method. We compared these two available materials to a new xenograft derived from cultivated coral. A higher processing technique applied to the bovine bone correlates with an increase in material crystallinity and a decrease in resorption rates during the osseous healing process. This correlation has been substantiated by findings from Amid et al.^21^ and Abdelmoneim et al.^22^. Moreover, prior clinical research has confirmed that xenografts processed at high temperatures and low-temperature processing xenografts exhibit similar efficacy in promoting new bone formation^20,23^. The structure and composition of Coral’s skeleton closely resemble that of natural bone^13–15^. PCM particles possess osteoconductive and resorption properties, making them ideal for bone augmentation procedures^13^. Additionally, PCM does not induce inflammatory infiltrates or fibrous encapsulation^24,25^. To assess the efficacy of new graft materials, we have created a new Micro-CT model that can identify both new bone formation and graft residuals.

## Results

There was no wound infection or morbidity in any of the animals in this study, and the 8 rabbits survived the experimental procedure.

### Micro-CT: bone volume analysis

**Figure 1:**
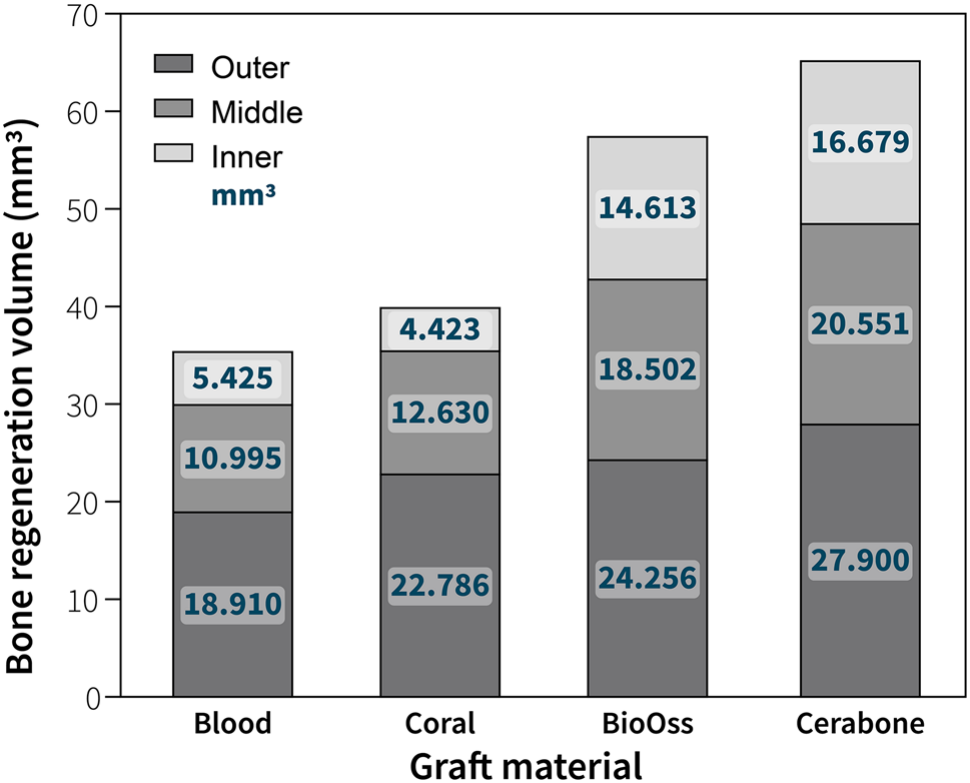
The volume of the new bone formed per region.

**Figure 2:**
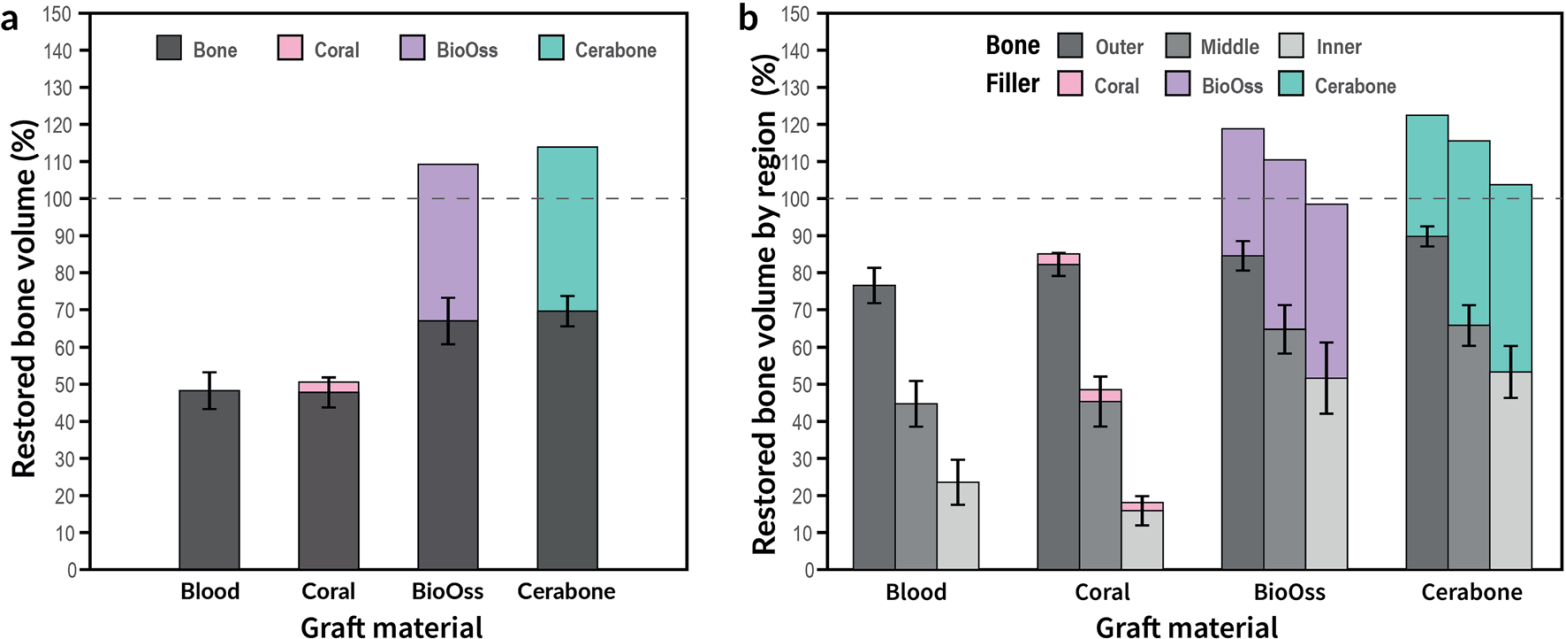
(**a**) Total restored bone and graft material volumes as a percentage compared to the pristine bone. (**b**) Restored bone volume by region as a percentage compared to the pristine bone.

In general, all the grafting materials showed the same pattern of bone formation, regardless of the material used. The volume of new bone formation was lowest in the center of the defects (i.e., inner), indicating that new bone formation declined as it progressed inward (Fig. 1). New bone formation within the defects did not reach the volume of the pristine bone (Fig. 2A,B, Table 1). The middle regions of the defects showed statistically significant differences between the grafting materials (one-way ANOVA; p = 0.038), whereas the measured volumes of the newly formed bone in the periphery (i.e., outer) of each defect did not (p = 0.156) (Fig. 1, Table 1). New bone formation was significantly different between the grafting materials only in the inner parts of the defects (p = 0.001). According to Tukey’s post hoc analysis, when the defects were filled with blood clot the measured percentage of the newly formed bone was lower compared with the DBBMb or DBBMc (p = 0.048 or p = 0.034, respectively). In addition, when the defects were filled with PCM, the new bone formation was lower compared with the defects filled with DBBMb or DBBMc (p = 0.008 or p = 0.005, respectively) (Table 1).

**Table 1:** The percentage of new bone and graft material per region: micro CT results.

	Percentage of bone regeneration per region = region volume/pristine bone volume			Percentage of graft material volume out of total volume per region = graft material/(bone + graft material)		
	Inner	Middle	Outer	Inner	Middle	Outer
	Mean (%) ± SD	Mean (%) ± SD	Mean (%) ± SD	Mean (%) ± SD	Mean (%) ± SD	Mean (%) ± SD
Blood	23.57± 18.01	44.72±18.30	76.54 ± 14.29	0.00 ± 0.00	0.00± 0.00	0.00± 0.00
PCM	15.88±12.00	45.34± 19.89	82.23± 9.60	15.07 ± 8.08	7.24± 3.65	3.32± 1.73
DBBMb	51.67±27.98	64.77± 19.18	84.55± 12.07	50.03 ± 14.50	40.92± 8.26	28.21± 8.34
DBBMc	53.32±20.62	65.80± 16.20	89.79± 8.44	49.53 ± 13.31	42.79± 10.52	25.54± 11.49
one-way ANOVA	p = 0.001	p = 0.038	p = 0.156	p<0.001	p<0.001	p<0.001
Post hoc analysis (p-value<0.05)	Blood<DBBMb	Post hoc was not significant		Blood<DBBMb		
	Blood<DBBMc			Blood<DBBMc		
	PCM<DBBMb			PCM<DBBMb		
	PCM<DBBMc			PCM<DBBMc		

Comparison between the different parts within the defects (i.e., inner/middle/outer): paired T-test analysis showed that regardless of the graft used, there were statistically significant differences between the various parts (p<0.05), except for the middle and the inner parts of the defects in the DBBMb group (p = 0.059 for the bone tissue and p = 0.053 for the graft material residual).

### Histometric analysis

**Figure 3:**
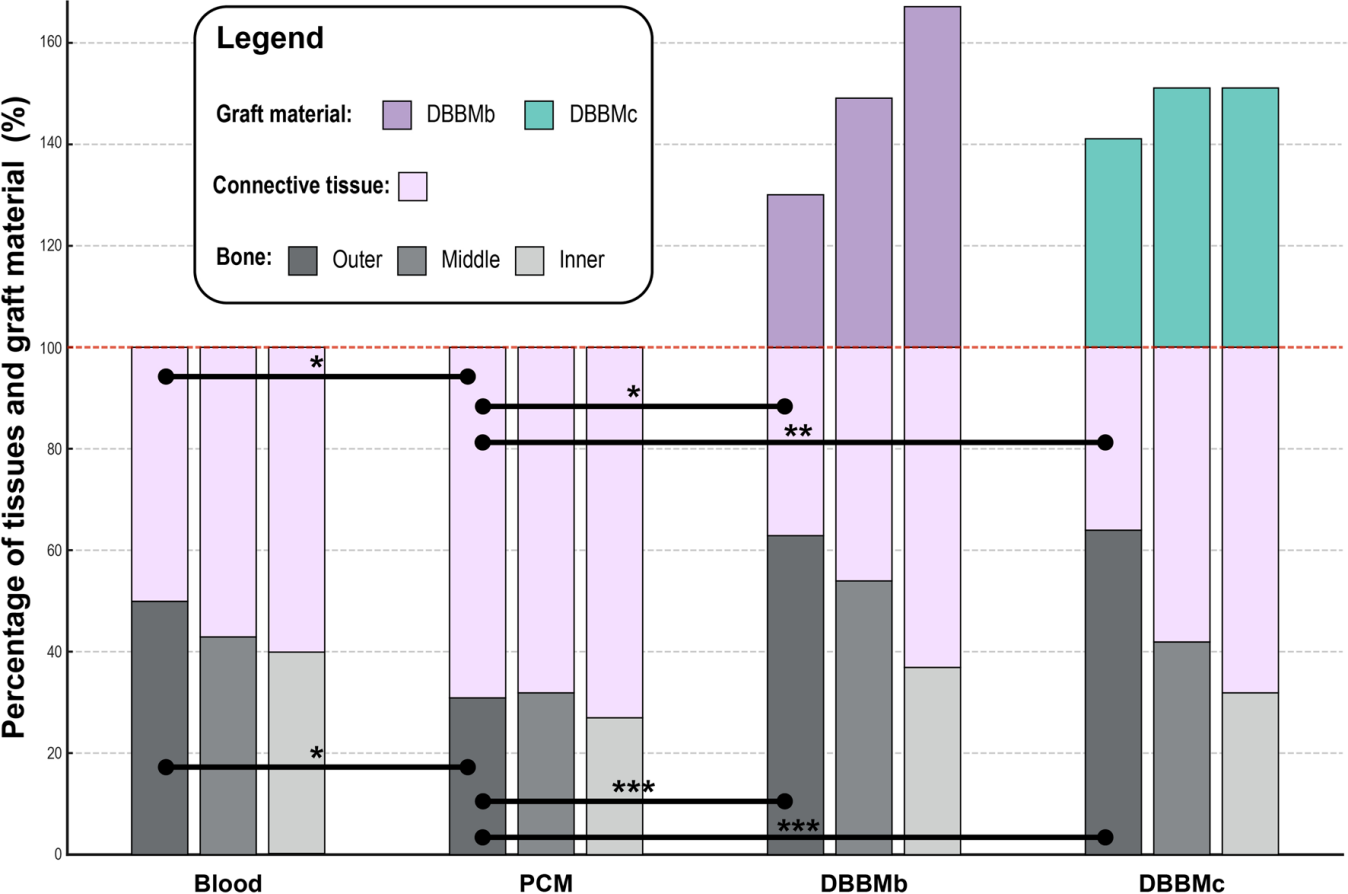
Regional distribution of connective tissue, bone, and graft material: results from histomorphometric analysis. Only statistically significant differences in corresponding regions are shown (e.g., comparisons are made between outer to outer, middle to middle regions, etc.).

The findings from our study reveal a consistency between histological observations and micro-CT analysis. A trend is observed as we examine closer to the center of the defects, characterized by reduced bone formation and increased formation of connective tissue. Notably, the PCM graft was entirely resorbed, leaving no detectable residues in the histomorphometric analysis (Fig. 3, Table 2). This outcome is similar to that of the blood clot defect group, which did not utilize any graft material. However, it is important to note that the defects treated with PCM exhibited a significantly higher proportion of connective tissue compared to those treated with DBBMb and DBBMc across all sections, as well as the blood clot group in the outer regions (Fig. 3, Table 2). A statistically significant difference was observed in the outer parts among the different materials. It is noteworthy that there was no difference between DBBMb and DBBMc.

**Table 2:** The average percentage of connective tissue, bone, and graft material per region of the histomorphometry of the defect is shown as a mean value with standard deviation (± SD).

	% of the connective tissue per region Connective tissue/(connective tissue + bone tissue + graft material)			% of the bone tissue per region Bone tissue/(connective tissue + bone tissue + graft material)			% of the graft material per region Graft material/(connective tissue + bone tissue + graft material)		
	Inner	Middle	Outer	Inner	Middle	Outer	Inner	Middle	Outer
	Mean (%) ± SD	Mean (%) ± SD	Mean (%) ± SD	Mean (%) ± SD	Mean (%) ± SD	Mean (%) ± SD	Mean (%) ± SD	Mean (%) ± SD	Mean (%) ± SD
Blood (n = 8)	60.39 ± 19.38	56.66 ±23.72	50.45 ±14.32	39.61 ±19.38	43.34 ± 23.72	49.55 ± 14.32			
PCM (n = 8)	73.03 ± 17.31	67.54 ± 15.06	69.46 ± 12.99	26.98 ± 17.31	32.46 ± 15.06	30.54 ±12.99			
DBBMb (n = 7)	63.28 ± 14.69	46.08 ± 13.73	36.73 ± 6.90	36.72 ± 14.69	53.92 ± 13.73	63.27 ± 6.90	67.44 ± 52.25	49.36 ± 30.12	30.13 ± 16.71
DBBMc (n = 7)	67.84 ± 17.36	57.87 ± 17.29	35.58 ± 15.90	32.16 ± 17.36	42.13 ± 17.29	64.42 ± 15.9	51.44 ± 17.45	50.87 ± 21.39	41.38 ± 17.94
one-way ANOVA*	p = 0.5060	p = 0.1778	p<0.0001	p = 0.0506	p = 0.1778	p<0.0001	p = 0.4572	p = 0.9160	p = 0.2480
Post hoc analysis (p-value<0.05)	ns	ns	PCM>Blood	ns	ns	Blood>PCM	ns	ns	ns
			PCM>DBBMb			DBBMb>PCM			
			PCM>DBBMc			DBBMc>PCM			

*t test was used for the graft material, since only two groups were compared.

## Discussion

In this study, we developed a novel Micro-CT model to analyze the regenerative potential of three distinct xenografts, compared to decalcified histomorphometry in surgically-created calvaria bone defects in rabbits. In general, Micro-CT analysis is less skill-dependent than the production and analysis of the histological slides since it is non-invasive, non-destructive, precise, and reproducible. Moreover, the invasive and irreversible techniques used to prepare the sections for histology might potentially affect the results^26,27^.

The new Micro-CT model allowed us to divide each defect into four equal cylindrical parts after segmenting out the remaining graft material. Thus, it provided a three-dimensional, more precise quantification and better understanding of the process of bone formation in CSD. Indeed, the results indicated a statistically significant differences between the grafting materials in the inner parts of the defects, whereas the amount of new bone formation was similar in the peripheral and middle parts.

Spontaneous bone formation in the peripheral part of the defect after 8 weeks can be expected^28^, however, not in the inner area of the defect^7^. Therefore, the region of interest should be the inner part of the defect that will better indicate differences between various grafts. Separating the inner part of the defect from the other parts allows an accurate observation of the healing process, as it is the most challenging area for healing, since it is remote from the origin of the migrating cells. Providing an insight into the inner region can better predict the regenerative potential of the bone augmentation graft material. Utilizing the surrounding intact bone as a control in the proposed model optimizes the use of the animal. Given the small size of the calvaria in rodent species, employing the intact bone as a control facilitates additional room for experimental defects. Moreover, this approach enables each defect to be assessed in comparison with the adjacent normal bone.

**Figure 4:**
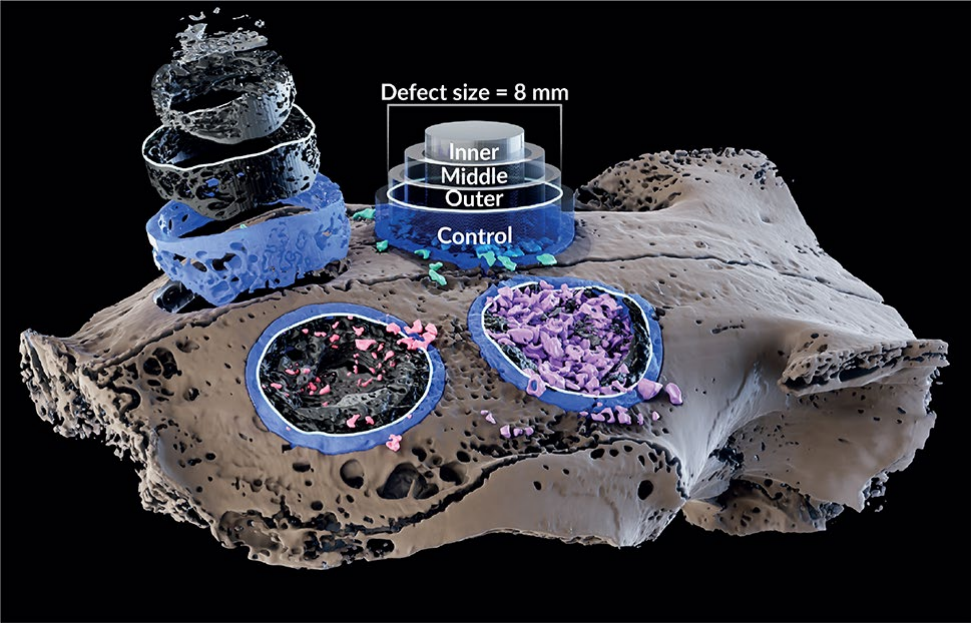
3D model of the rabbit calvaria presenting the segmentation and the quantification method of the four 8 mm critical size defects: light brown—calvaria; segmented graft material particles: light green—DBBMb; light purple—DBBMc; pink—PCM. In order to partition the ROI into four equal concentrical volumes for subsequent comparison, each defect was virtually separated using cylinders modelled using a formula of a cylinder with varying radii (i.e., $V_{Inner} = V_{Middle} = V_{Outer} = V_{Control}$ ). Top left: the unfilled defect showing the subdivided ROI of the bone, virtually separated on a Z-axis.

Amongst the various advantages of the Micro-CT, it allows to virtually segment out even the smallest traces of the graft material and evaluate only the newly formed bone. In our current research, we demonstrated that the evaluation of the defect augmented with PCM using three-dimensional Micro-CT revealed the presence of numerous particles that were not resorbed (Figs. 2 and 4, and Table 1. Conversely, two-dimensional histological analysis was unable to detect this detail (Fig. 3, Table 2).

It has been suggested that the same biomaterial may exhibit varying performance in new bone formation, depending on the manufacturing processes utilized^20^. Cook and Mealey^29^ compared two xenograft biomaterials and found a statistical difference in terms of the percentage of bone formation in histomorphometric parameters. However, there was no significant difference in the percentage of new bone formation among bone substitute biomaterials that come from the same source. Although there may be some variations in their biological behavior, these differences do not affect the amount of new bone formation following a sufficient amount of healing time^30^.This biomaterial has a slow resorption process, which has two interpretations. Firstly, it could maintain socket dimensions after tooth extraction^31^ On the other hand, the amount of remaining graft material could negatively affect osseointegration success^32^.

Autologous bone grafting is widely accepted as the gold standard for bone grafting due to its proven effectiveness. However, this procedure is not without limitations. Autologous bone grafts tend to resorb quickly, which can lead to complications, also, the procedure can result in notable morbidity^33,34^. The aim of our study was to evaluate the performance of xenografts as a standalone material for bone grafting. Although combinations of xenografts with autogenous bone have shown favorable results in various cases, our research focused on assessing the performance of xenografts independently. Our findings may provide valuable insights into the efficacy of using xenografts as a standalone material for bone grafting.

In this model, the PCM was compared to two bovine xenografts: DBBMb, DBBMc; and a spontaneous blood clot. When the defects were filled with blood clot, the newly formed bone volume percentages were lower compared to the DBBMb and DBBMc groups, similar results were found for the PCM.

Histological analysis often does not reveal evidence of remnants of PCM after two months^35^. Yet, in our study by using the Micro-CT, we were able to trace some PCM residuals, indicating that the material was almost fully resorbed but not completely. This finding could only be achieved by utilizing the three-dimensional perspective of the Micro-CT.

Grafting materials may affect the amount of regenerated bone tissue, and their presence may alter the microarchitecture of newly formed tissue. It appears that a grafting material’s ability to aid bone regeneration, as well as the resorption rate of the bone, have an effect on both bone healing mechanism and the morphology of the newly constructed tissue. Such differences might affect the overall quality of the newly-formed bone^10^.

It was suggested that residual biomaterials could interfere with normal bone remodeling and healing, as significant variations in vital bone formation utilizing different grafting materials were found that possibly negatively affect the bone surrounding the implants in terms of quality and architecture^36^.

Moreover, the high-resolution scans provide better view on defect morphology, as bi-cortical defect and penetrating the dura mater can influence bone healing^37^. The ability to investigate the extent of a lesion in three dimensions allows us to determine whether the defect affects one or both cortical layers. Traditional histological analysis, which uses two-dimensional slices, is limited since each slice provides only a narrow view of the region of interest. The Micro-CT scan, however, allows us to measure volumes in any desired orientation.

In a study by Halperin-Sternfeld et al.^38^ Micro-CT was used to explore the advantages of an hydrogel-filled defects in rat model. The Micro-CT results indicated bone regeneration from the defect margins inwards, yet, bony islets were observed in the center of the defect (i.e., inner region). Sohn et al.^39^ claims that the dura mater and the periosteum have an osteogenic potential, which can explain the formation of bony islets away from the defect margin^39^. It appears that this phenomenon is more prevalent in noncompliant bi-cortical defects.

It should be noted that our study has a limitation in the method used for histological analysis. We utilized decalcified histology, but did not perform histochemical analyses. This limitation affects the visualization and interpretation of certain cellular structures and dynamics within the samples. Furthermore, the lack of calcified histology in this study represents a potential limitation, as it may have impacted the ability to perform further comparative analysis.

### Conclusions

In conclusion, the new Micro-CT model allowed us to divide each defect into four cylindrical parts of equal volume after segmenting out the remaining graft material. This approach provided a three-dimensional, more precise quantification and a deeper understanding of the bone formation process. Notably, the results indicated statistically significant differences between the grafting materials in the inner parts of the defects, while the amount of new bone formation remained similar in the peripheral and middle regions. Consequently, it might be worthwhile to consider a similar approach for histological analysis, which would aim to distinguish between inner and outer regions. In this way, a more precise and accurate diagnosis can be made. One of the noteworthy advantages of Micro-CT is its capability to virtually isolate even the most minute remnants of the grafting material, enabling a specific assessment of newly formed bone. Our present study has demonstrated that when evaluating defects augmented with PCM using three-dimensional Micro-CT, numerous non-resorbed particles were detected. In contrast, conventional two-dimensional histological analysis failed to discern this level of detail.

## Methods

### Study sample

Eight adult female New Zealand white rabbits (3.5-4.0 kg) were subjected to calvarial bone defect model. The experimental procedures involving live vertebrates and/or higher invertebrates in this study were approved by the institutional committee of animal care and use (IACUC) of Tel Aviv University (no 01-18-071), and all experiments were conducted in strict accordance with the approved guidelines. Additionally, this study adhered to the ARRIVE guidelines for the reporting of animal experiments to ensure high standards of research quality and reproducibility. Animals were individually housed in the Central Animal Facility of the Tel Aviv University. Animals were fed with Teklad Global Rabbit Diet (Envigo, Madison, Wisconsin), autoclaved hay, and tap water *ad libitum*.

### Surgical procedures

Surgical procedure was performed under general anesthesia following sedation with 5 mg/kg subcutaneous (SC) xylazine (Sedaxylan Veterinary, Eurovet Animal Health BV Bladel, The Netherlands) and 35 mg/kg SC ketamine (Clorketam, Vetoquinol, Lure, France). Oxygen was delivered by means of a facemask connected to an anesthetic machine with an oxygen flowmeter (model Mix4; Foures SAS, Bordeux) at a rate of 2 l/min. Blood oxygen saturation, pulse and body temperature were monitored by a PhysioSuite monitor (Kent Scientific). Once anesthetized, the surgical area was shaved and disinfected with iodine solution and local anesthesia was administrated with 2% lidocaine hydrochloride and norepinephrine (1:100,000) for reduction of pain and hemostasis. The rabbit calvarium were exposed via a midsagittal longitudinal incision.

**Figure 5:**
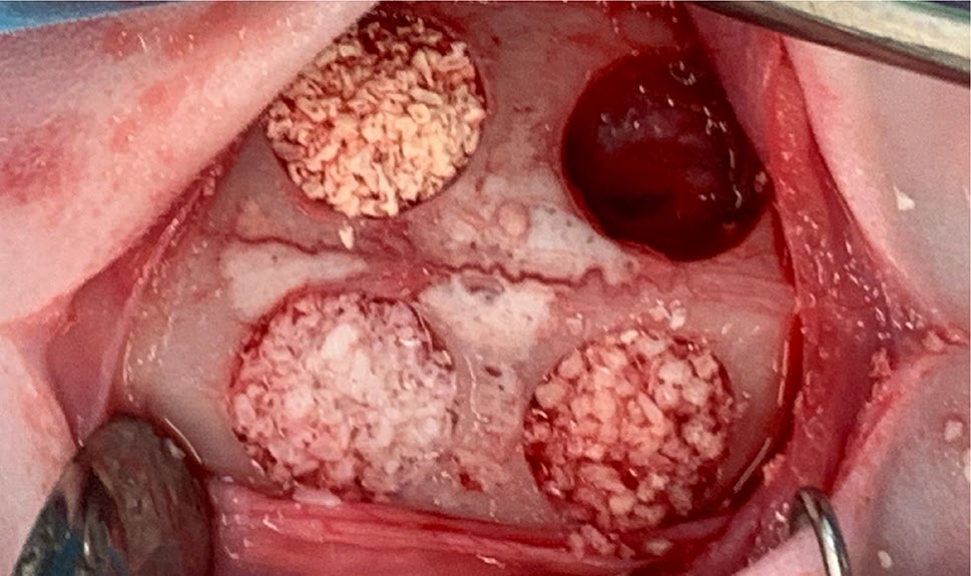
The surgical procedure showing four defects of 8 mm size grafted with three xenografts and the fourth served as a spontaneous blood clot control.

The periosteum was carefully separated from the bone and skin flaps were elevated exposing the calvarium. Four circular defects 8-mm in diameter were prepared in the bone under copious water irrigation (Fig. 5). To mark the dimensions of the defects, an 8-mm trephine bur was used, followed by a gentle bone removal process using an inverted conical diamond bur. The surgical procedure was conducted by Dr. Beitlitum Ilan and Dr. Omer Cohen. Special attention was given to avoid damaging the dura mater. Random allocation of the four treatment modalities was done for all 32 defects, with four defects per animal:

1. Pure Coral Mineral Particles (PCM), a mixture of particles produced from two different coral species of the Acropora coral family - in the form of Argagonit pure mineral part of the coral enriched by inorganic ions, predominantly silicium (CORBONE®, Misgav, Israel), small particles ranging from 0.5 to 1 mm.
2. Deproteinized Bovine Bone Mineral - Bio-Oss (DBBMb) (Geistlich Pharma AG, Wolhusen, Switzerland), small granules ranging from 0.25 to 1 mm.
3. Deproteinized Bovine Bone Mineral - Cerabone (DBBMc) (Botiss Biomaterials, Berlin, Germany), small particles ranging from 0.5 to 1.0 mm.
4. Control - Defect was left empty until filled with blood from spontaneous bleeding.

For grafting the experimental defects, we employed a 1 cc disposable syringe, marked with 0.1 cc graduations, to ensure precise measurement. The tip of the syringe was removed, and it was then loaded with the biomaterial, which had been pre-soaked in sterile saline solution. Subsequently, we carefully placed a measured quantity of 0.1 cc of the wet biomaterial into each defect.

The defects were covered with pericardium-derived collagen membrane (JASSON, Botiss Biomaterials, Berlin, Germany). The periosteum was sutured and stabilized by a horizontal internal mattress 5-0 coated PGA (Omnia, Fidenza, Italy). This was followed by dermis closure using 9 mm autoclips®(MikRon®Precision Inc., Gardena, CA) for a primary wound closure. Postoperatively, the animals received medication for pain relief and infection control for 3 days, which included Enrofloxacin (Baytril®, Bayer, USA) 5mg/kg/day, and Carprofen (Carprieve®, Norbook, Ireland) 5 mg/kg/day. The animals were monitored and weighed weekly to detect post-operative complications or deterioration. Animals were euthanized after 8 weeks by a combination of xylazine and ketamine SC followed by a lethal dose (30 mg/kg) of pentobarbitone IV (CTS, Pharmaceutical Industries Inc., Kiryat Malachi, Israel). After euthanization all specimens were fixed in 10% neutral buffered formalin for 76 hours and surgical calvarial sites were retrieved en bloc and prepared for Micro-CT evaluation.

**Figure 6:**
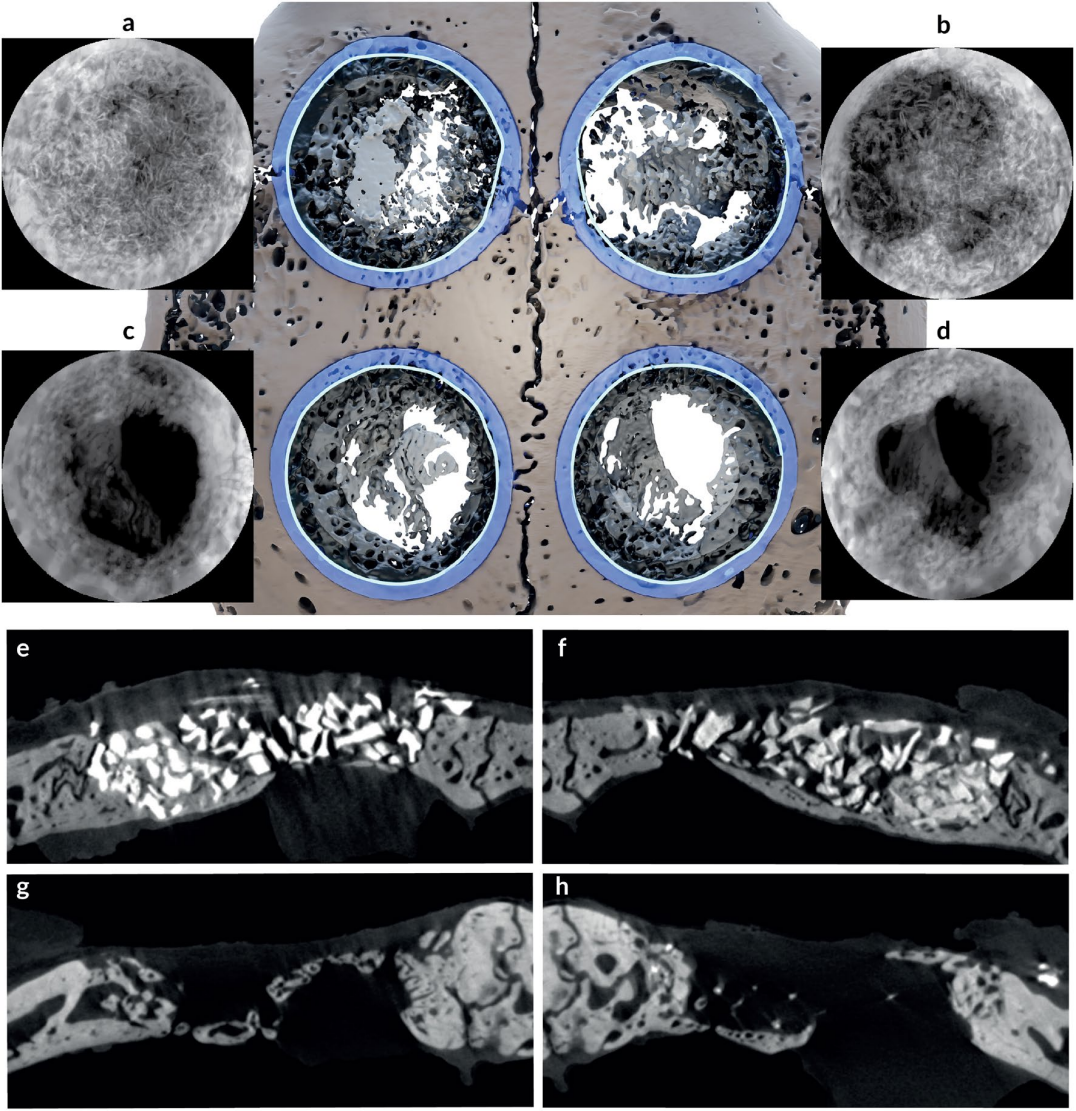
Top (**a**–**d**): Dorsal view of the 3D model of the rabbit calvarial bone presenting with four defects. Each defect was shown as an x-ray projection of the bone from the occlusal view following the segmentation of the bone from the graft materials: (**a**) DBBMc; (**b**) DBBMb; (**c**) control; and (**d**) PCM. Bottom (**e**–**h**): Coronal cross-sections of the Micro-CT scan through the middle of each defect exhibiting the particles of the graft material amongst the islands of the newly formed bone: (**e**) DBBMc; (**f**) DBBMb; (**g**) control; and (**h**) PCM.

### Micro-CT analysis

All calvarium were scanned using Micro-CT (XT H 225 ST, Nikon Metrology NV, Leuven, Belgium), equipped with a 225 kV 225 W reflection target, housed at the Shmunis Family Anthropology Institute, Dan David Center for Human Evolution and Biohistory Research, Sackler Faculty of Medicine, Tel Aviv University. Scans were performed at an isotropic resolution of 23 $\mu$m utilizing the following parameters: 160 kVp energy, at 138 $\mu$A intensity, and with 3141 projections utilizing a 354 msec exposure time. Raw scans underwent reconstruction procedure in Nikon CT Pro 3D software (v. 6.9.1; Nikon Metrology NV, Leuven, Belgium) and subsequent segmentation using various semiautomatic tools based on grayscale thresholds with appropriate manual refinement prior to the analysis in Amira software (v. 6.3, www.fei.com). Each scan was segmented into bone, and three types of graft materials: DBBMb, DBBMc, and PCM. In all samples the graft material particles were clearly visible and morphologically distinguishable from the surrounding bone (Fig. 6). Circular regions of interest (ROI) of 8 mm diameter were identified via the surgical borders and subject to the volumetric analysis. Each 8 mm circular defect was virtually divided using three coaxial 3D cylinders of varying radii (modelled to produce a central cylinder and two tubes of equal volumes) into three regions (outer, middle, and inner) by intersection with the segmented bone and graft volumes. A fourth cylinder of a larger diameter and equal volume was created around each ROI to serve as a reference for a normal bone thickness (Fig. 4). Volumes of the restored bone were calculated as a ratio of the bone volume present in each of the three regions to the control volume of the surrounding pristine bone that served as control in the same defect (Fig. 4).

### Histometric analysis

Following fixation in 10% neutral buffered formalin for 76 hours, tissue samples underwent decalcification in 5% formic acid over a period of 14 days, and subsequently were encased in paraffin. To conduct the histological examination, the three central sections, each measuring 5 micrometers in thickness, were chosen and subjected to staining using hematoxylin and eosin (H &E). Photographs of the stained samples were obtained using a TCA-3 digital camera (Tucsen Imaging Technology Co, Jiayijie Province, Shenzhen, Guangdong, China) attached to a light microscope (Olympus, Tokyo, Japan) at 40X magnification. Subsequently, these images were segmented using Adobe Photoshop (Adobe system software, Riverwalk, Citywest Business Park, Dublin, Ireland) facilitating the differentiation of bone, connective tissue, and graft material. Prior to measurement, the system underwent calibration and image digitalization, utilizing Bioquant Osteo 2009 version 9XP software (Bioquant Image Analysis Corporation, Nashville, Tennessee) for precise assessment. Areas showcasing new bone formation and soft tissue were meticulously outlined to highlight specific areas of interest. The edges of each 8 mm defect were identified and the defect was divided into three zones, progressing from the outer edges towards the center: outer, middle, and inner, similarly to the Micro-CT analysis. The surface area of bone, connective tissues, and graft material was calculated for each slide, enabling comparison within and between the four groups. Graft material residues were observed only in the slides of two groups (DBBMb and DBBMc). To enable comparisons among all groups, the percentages of bone and connective tissue were calculated using the following formulas: bone (%) = bone/(bone + connective tissue), and connective tissue (%) = connective tissue/(bone + connective tissue). The percentage of graft material remnants was represented as graft material/(bone + connective tissue). The analysis was conducted using one-way ANOVA and paired t-tests in Graphpad Prism software (v. 8.0.1; GraphPad Software Inc.; San Diego, CA, USA).

## Data Availability

The datasets used and/or analyses during the current study available from the corresponding author on reasonable request.
